# Minimally Invasive Endoscopic Spine Surgery as a Viable Option for Geriatric Patients with High Anesthetic Risk

**DOI:** 10.7759/cureus.71886

**Published:** 2024-10-19

**Authors:** Cameron Myers, Helena Krogman, Bryan Stevens, Sanjeev Kumar, Matthew Meroney

**Affiliations:** 1 Department of Anesthesiology, University of Florida College of Medicine, Gainesville, USA; 2 Department of Pain Management, University of Florida College of Medicine, Gainesville, USA

**Keywords:** chronic pain, endoscopic spine surgery, geriatric medicine, lumbar decompression, minimally invasive surgery

## Abstract

Spinal stenosis poses a significant healthcare challenge in the United States. This case report delineates the progressive interventions in a male over 90 years of age with lumbar spinal stenosis, emphasizing the shift toward minimally invasive endoscopic spine surgery. The patient, burdened by neurogenic claudication, failed conservative measures, leading to substantial pain and diminished quality of life. Traditional open spine surgery, fraught with morbidity and mortality risks, prompted consideration of alternative approaches. The case details the journey of the patient through various interventions, culminating in endoscopic decompression as opposed to more invasive open surgical methods. It is important to note that the patient was evaluated by colleagues in the facility’s neurosurgical department and deemed not a candidate for surgery due to his age and comorbidities. The elderly patient with a complex medical history underwent a myriad of marginally effective treatments, prompting the ultimate intervention, a minimally invasive interlaminar endoscopic decompression. Postoperatively, the patient reported significant improvement in a subjective pain score based on 0 being no pain and 10 being debilitating pain, marking a paradigm shift from 10 out of 10 pain to an average of two out of 10. The discussion centers on the evolution from traditional open spine surgery to endoscopic procedures and factors to consider when deciding the appropriate therapy. The case underscores the favorable outcomes associated with endoscopic decompression and compares the recent literature to more traditional surgical operations. While acknowledging the limitations of endoscopic decompression, especially in challenging anatomical regions, the report emphasizes the promising role of endoscopic procedures, particularly in high-risk populations. The geriatric patient, refractory to conventional treatments, achieved excellent pain relief without reported sequelae from open surgical techniques.

## Introduction

Spinal stenosis is a prevalent orthopedic condition that significantly impacts a large segment of the population, particularly older adults. It involves the narrowing of spaces within the spine, which can lead to compression of the spinal cord and nerves. The following case report illustrates progressive interventions in a male patient over 90 years of age with neurogenic claudication due to lumbar spinal stenosis. Despite conservative treatments, the patient continued to experience significant pain adversely affecting his quality of life. Traditional open spinal surgery carries considerable morbidity and mortality risks, particularly in vulnerable populations such as the elderly and those with multiple comorbidities. Key considerations for patients undergoing more invasive open spine surgery include musculoskeletal instability necessitating further intervention, longer hospital stays, increased postoperative pain compared to endoscopic approaches, and extended clinical follow-up periods [1]. This case report centers on a patient with elevated risk factors who chose endoscopic decompression, resulting in significant pain relief and improved quality of life, in contrast to the more invasive open surgical methods considered.

## Case presentation

A male patient over 90 years old, with a medical history of multivessel coronary artery disease, benign prostatic hyperplasia, chronic obstructive pulmonary disease, essential hypertension, hyperlipidemia, obstructive sleep apnea, and osteoarthritis, presented with chronic lumbar radicular back pain that began after his service in the Korean War. He reported increased pain during ambulation, sitting, standing, bending forward, and lifting, as well as with walking and light exercise, severely affecting his quality of life and ability to perform daily activities. His symptoms showed minimal improvement with rest but significantly improved with forward flexion and sitting, consistent with a diagnosis of neurogenic claudication. The pain radiated down both lower extremities, accompanied by numbness without changes in bowel or bladder activity. Lumbar MRI revealed multi-level central and neuroforaminal stenosis, most severe at the L4-5 vertebral level.

Approximately one and a half years before his current presentation, the patient attempted physical therapy and acupuncture without resolution of symptoms. He also underwent trials of oral pain medications including acetaminophen, non-steroidal anti-inflammatory drugs, gabapentin, opioids, and selective serotonin-norepinephrine reuptake inhibitors, each for more than six weeks with minimal improvement. Subsequently, he underwent bilateral percutaneous lumbar decompression of L2-3 and L4-5, specifically the minimally invasive lumbar decompression (MILD) procedure. Three months after the MILD procedure, he experienced near-complete resolution of right-sided pain but continued to have persistent left-sided pain. Four months post MILD, he underwent lumbar medial branch blocks, sacroiliac joint injections, and Bertolotti's radiofrequency ablation over several months, resulting in only mild improvement in functional status and marginally increased mobility.

Despite these interventions, his left-sided lower back pain remained refractory. Before the interlaminar endoscopic laminotomy, he reported pain levels of 10 out of 10 on average on a numerical rating pain score of 0-10 and six out of 10 consistently. From the initial anesthesia encounter to discharge from the hospital, the hospital stay lasted 10 hours and 21 minutes, and the patient experienced an uneventful postoperative course. Ultimately, the patient underwent an interlaminar endoscopic minimally invasive procedure involving laminotomy, facetectomy, lateral recess, and L5 nerve decompression under direct endoscopic visualization. Immediately after the surgery, the patient reported significant improvement in his radicular pain.

**Figure 1:**
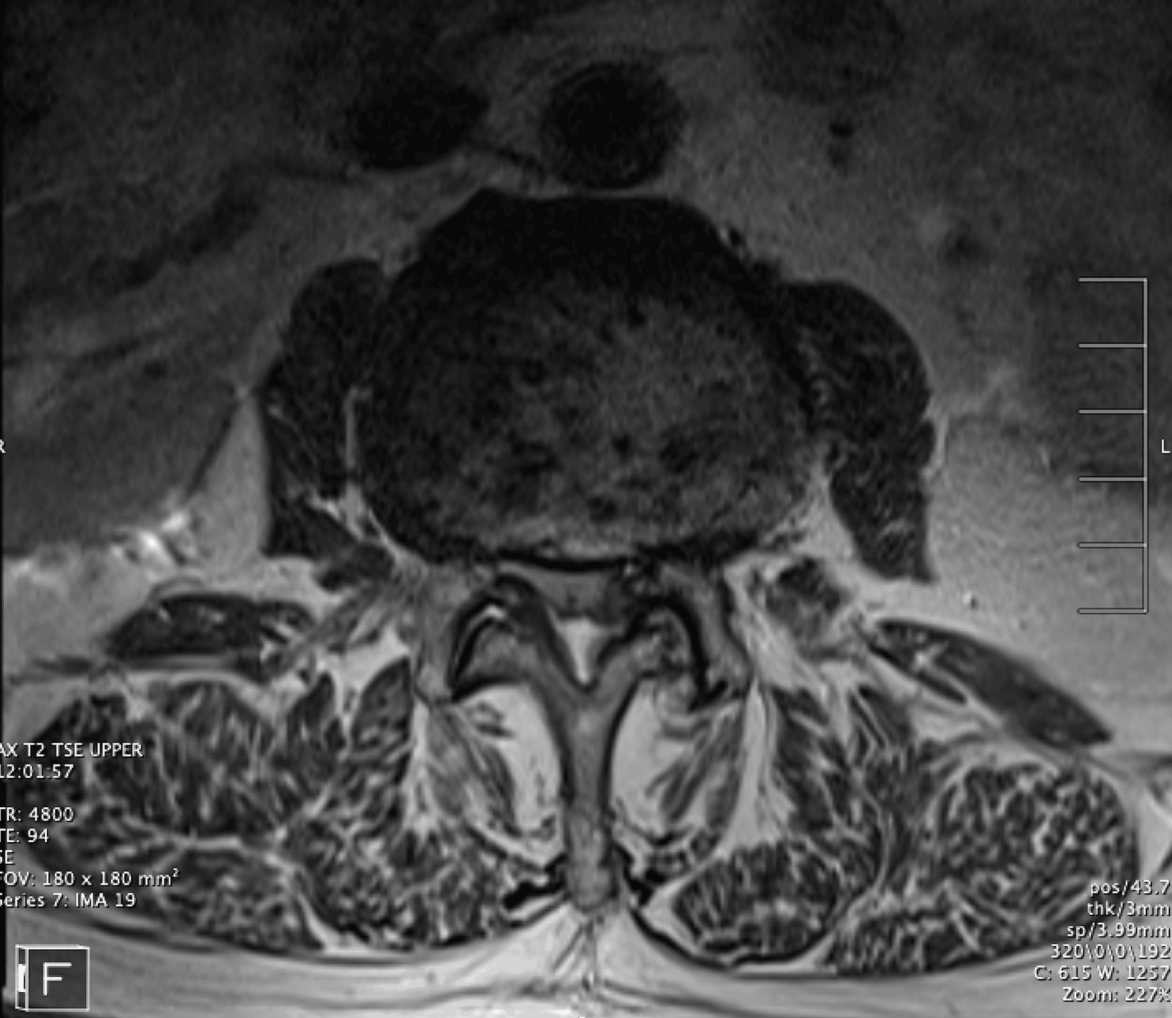
Axial lumbar MRI T2 This figure provides a detailed axial snapshot of the lumbar L4-L5 intervertebral level, highlighting the following pathological findings: posterior disc uncovering with a prominent circumferential disc bulge, particularly more pronounced on the left side; advanced facet arthropathy with joint effusions; moderate thickening of the ligamentum flavum; severe canal stenosis resulting in complete effacement of cerebrospinal fluid (CSF) around the nerve roots and in both lateral recesses; moderate right foraminal stenosis; and severe left foraminal stenosis.

**Figure 2:**
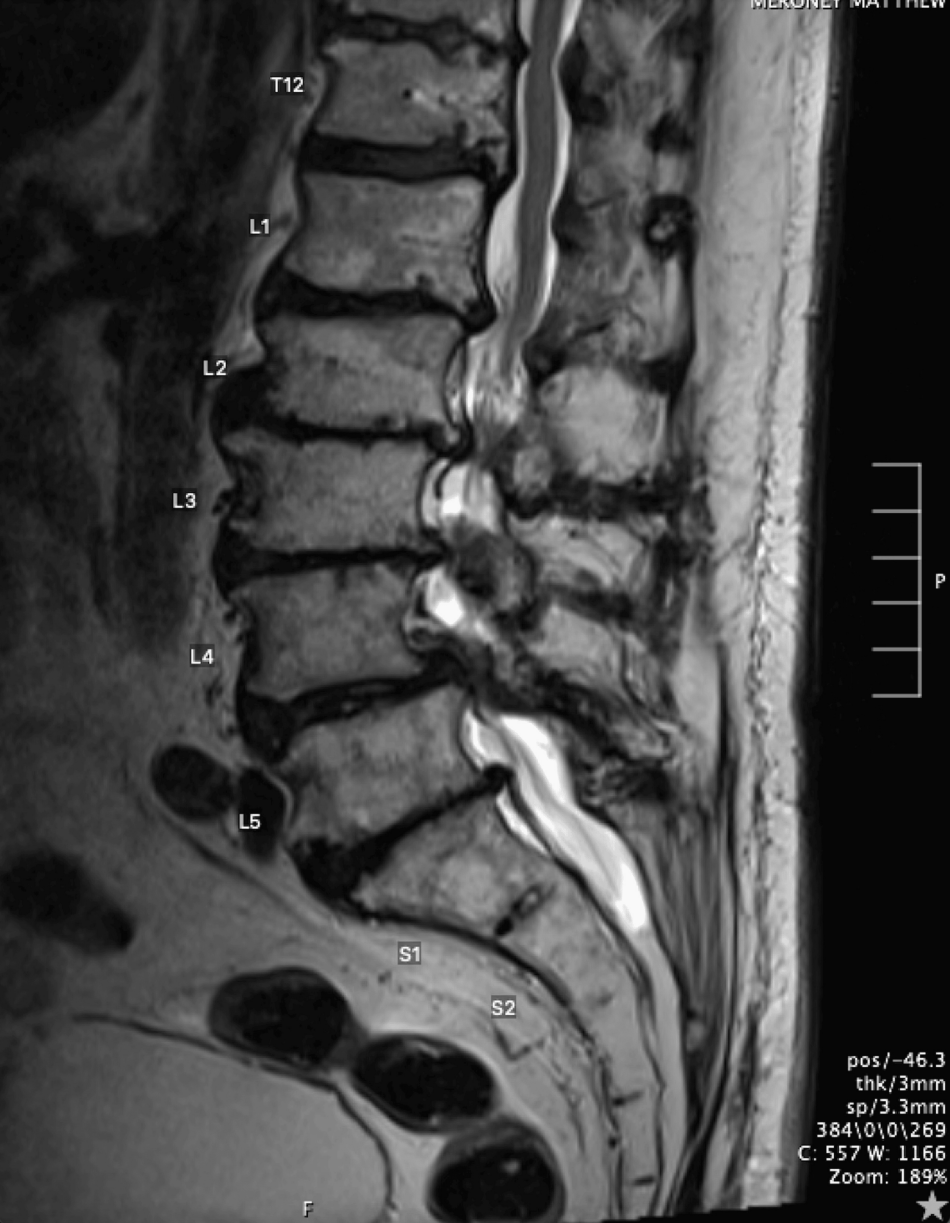
Sagittal lumbar MRI T2 This figure provides a detailed view of the sagittal lumbar spine, illustrating the following pathological conditions: advanced multilevel osteochondrosis, facet arthropathy, and interspinous pseudarthrosis throughout the lumbar region. Particularly notable are severe canal stenosis at the L2-L3 and L4-L5 levels. Additionally, there is partially visualized canal stenosis at T11-12, along with deformity of the spinal cord in this region.

**Figure 3:**
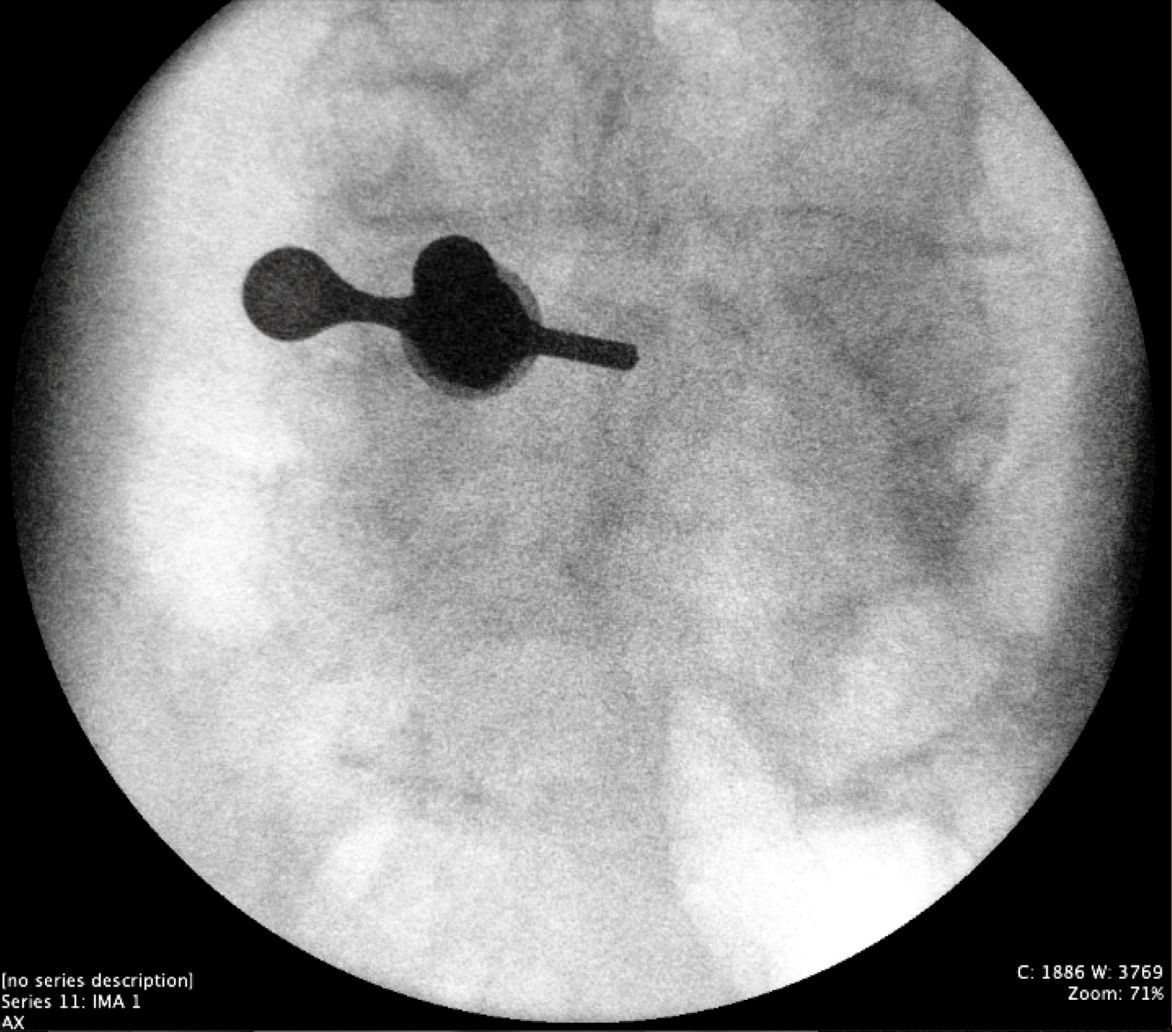
Anterior/posterior intraoperative fluoroscopy of the L4/L5 interlaminar window This figure offers a fluoroscopic view of the surgical instrumentation, showcasing the endoscope with its endoscopic port and working tube precisely positioned at the L4/L5 interlaminar window. The field of view is strategically centered, spanning both the descending lamina of L4 and the interlaminar window of L4-L5.

**Figure 4:**
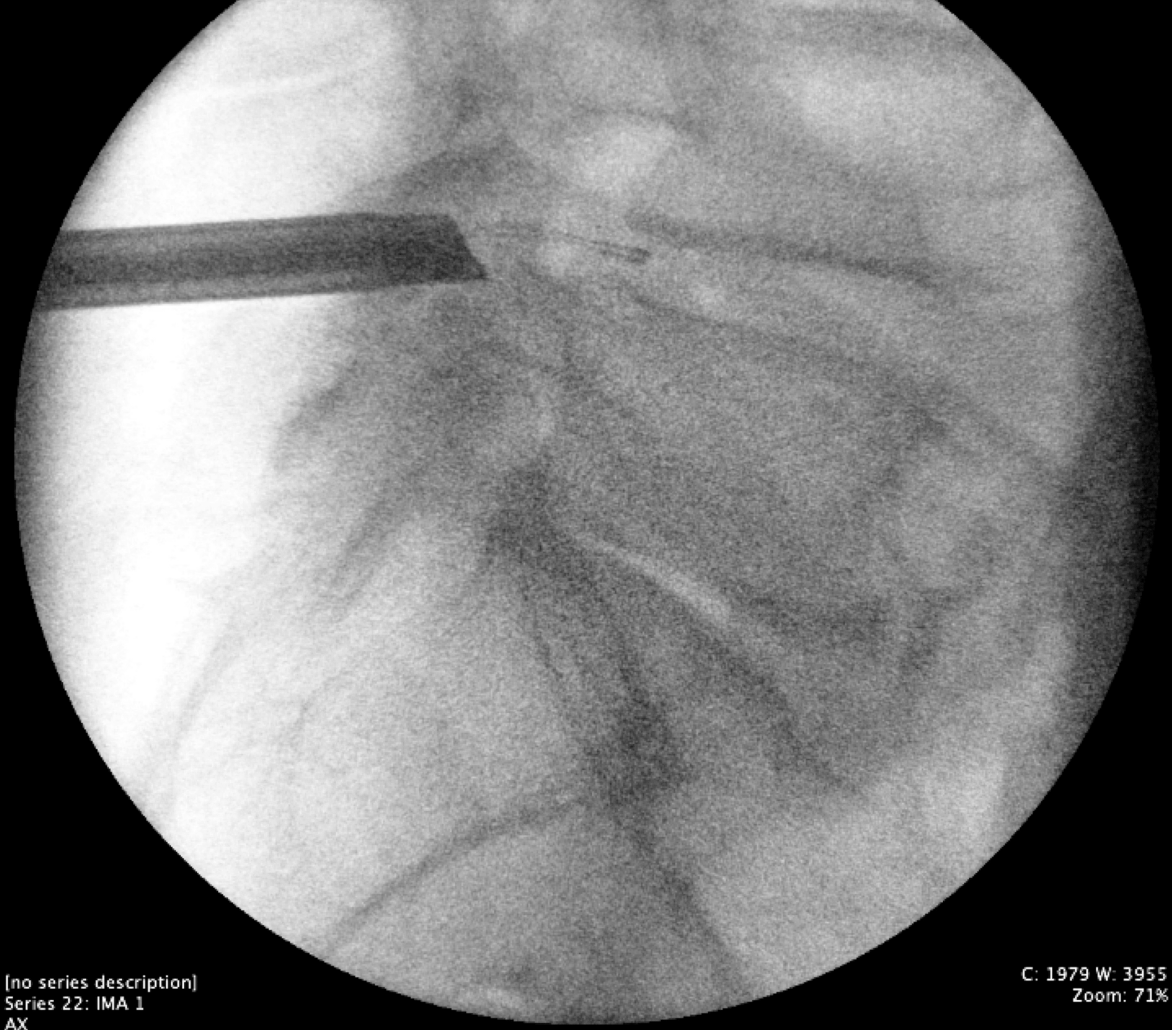
Lateral intraoperative fluoroscopy of the L4/5 interlaminar window Lateral intraoperative fluoroscopy depicting the electrocautery surgical instrument, known by its proprietary name Trigger Flex, created by Elliquence based in New York, extending into the L4-L5 disc space. This placement indicates successful surgical navigation to the anterior causative pathology.

Figures 1-4 collectively underscore the importance and effectiveness of the endoscopic technique in the management of complex lumbar spine pathologies. The axial snapshot (Figure 1) portrays pathological features at the L4-L5 intervertebral level, highlighting the suitability of endoscopy for detailed anatomical visualization and precise intervention. The sagittal view (Figure 2) further details the capability of endoscopic procedures in addressing advanced osteochondrosis, facet arthropathy, and severe canal stenosis across multiple lumbar segments. The fluoroscopic depiction (Figure 3) showcases the strategic placement of endoscopic instruments at the L4/L5 interlaminar window, essential for minimally invasive yet targeted treatments. Lastly, intraoperative fluoroscopy (Figure 4) underscores the successful navigation and utilization of the endoscopic approach, affirming its role in achieving optimal surgical outcomes while minimizing tissue disruption.

Together, these figures demonstrate the painting of a story that highlights the unique challenges found in every patient's pathologic variability. While the endpoint of decompression is still not universally described or agreed upon, it can certainly be considered as a prelude to more invasive surgeries that offer reduced recovery times and a lower overall complication rate, especially in the geriatric patient population.

At the one-week follow-up appointment after surgery, he reported significant improvement in his pain. He described his worst postoperative pain as three out of 10 on a numeric rating scale from 0 to 10 and an average pain level of two out of 10, a marked improvement from his pre-surgery pain level of 10 out of 10. He noted an 80% reduction in his baseline pain and can now walk for over 20 minutes without pain as compared to previously being unable to even stand. He reported no complications during the first week after surgery, and an examination of the surgical site revealed no signs of inflammation such as erythema, induration, drainage, warmth, or tenderness.

At his three-month follow-up, he reported complete resolution (100%) of his most severe pain complaint, characterized by debilitating sharp pain while standing or walking, which had previously caused suicidal thoughts. Overall, he reported more than 50% reduction in pain intensity. As of the latest update, six months after the procedure, the patient continues to experience substantial pain relief and has achieved an improved quality of life.

## Discussion

In the field of pain medicine, a cornerstone principle is to prioritize conservative measures for managing refractory pain conditions. However, when these prove insufficient for providing relief, clinical protocols often necessitate more invasive methods. Historically, traditional open spine surgeries have entailed soft tissue decompression, intervertebral disc dissection or debulking, and substantial removal of bony structures [1]. These procedures commonly result in undesired outcomes like facet joint destabilization and changes in adjacent spinal segments, requiring extensive and costly clinical management to address resultant anatomical alterations [2].

A recent meta-analysis comparing outcomes of open surgical interventions versus endoscopic spinal surgeries in patients with lumbar stenosis revealed comparable overall improvements in lumbar function following both lumbar fusion and endoscopic decompression, with comparable outcomes observed at 12 months [3,4]. However, the literature indicates that open surgeries often require longer postoperative recovery periods compared to minimally invasive endoscopic decompression approaches [2]. Endoscopic spinal surgery methods are increasingly favored due to reduced incidence of muscle injury, blood loss, and shorter overall operation times [5]. Notably, studies have shown that obese patients undergoing open procedures, such as discectomies, experience prolonged operative times compared to endoscopic techniques [6].

It is worth noting that this patient was initially considered inoperable by colleagues in the neurosurgery department due to advanced age and multiple comorbidities such as multiple coronary artery disease, chronic obstructive pulmonary disease, hypertension, and obstructive sleep apnea. Although several stenotic areas were identified that could have been surgically corrected, the most severely affected lumbar vertebral segment was chosen for intervention given the patient's high-risk profile. The patient achieved a satisfactory therapeutic outcome with no complications following endoscopic decompression. This success can be attributed to the minimally invasive nature of the approach, which allows for comparable pain relief with less disruption to supporting anatomical structures than traditional open surgery. Consequently, endoscopic procedures result in a reduced risk of damage to neural structures, epidural vasculature, and less extensive fibrosis during healing [2]. Additionally, in patients with severe osteoporosis, the risk of postoperative cage migration associated with open surgical techniques is mitigated with endoscopic decompression [1].

## Conclusions

Endoscopic procedures are emerging as a promising alternative for patients who would otherwise avoid open surgery due to their risk profile. While initial reports suggest higher costs associated with endoscopic decompression, recent studies indicate that it ultimately proves more cost-effective due to shorter hospital stays, better discharge outcomes, and reduced long-term follow-up requirements compared to traditional open-spine surgery. However, there are limitations to endoscopic decompression procedures. Accessing the cervical and upper thoracic vertebrae can be challenging due to anatomical constraints, and these areas pose higher risks when endoscopy is performed by less experienced physicians. The decision to opt for endoscopic versus open techniques must carefully consider factors such as the patient’s comorbidities, specific spine pathology, the expertise of the treating physician, and overall risk assessment, as with any surgical procedure. 

In this case study, despite being a high-risk geriatric patient with refractory and debilitating back pain, the endoscopic procedure was well tolerated and provided excellent pain relief without any reported sequelae that might have arisen from traditional open surgical techniques. The patient's positive outcome highlights the potential advantages of endoscopic techniques over traditional open surgeries. As minimally invasive procedures evolve, integrating these advancements into clinical practice promises to improve patient outcomes, reduce healthcare costs, and enhance the overall quality of life for individuals suffering from spinal disorders.
